# Perceived stress during the COVID-19 pandemic among the tunisian population

**DOI:** 10.1192/j.eurpsy.2021.770

**Published:** 2021-08-13

**Authors:** N. Regaieg, I. Baati, M. Elleuch, S. Hentati, J. Masmoudi

**Affiliations:** 1 Psychiatry “a” Department, Hedi Chaker UHC, Sfax, Tunisia, Sfax, Tunisia; 2 Psychiatry “a” Department, Hedi Chaker University Hospital, Sfax, Tunisia; 3 Psychiatrie “a” Department, Hedi Chaker Hospital University -Sfax - Tunisia, sfax, Tunisia

**Keywords:** perceived stress, COVID-19, PSS10, psychological stress

## Abstract

**Introduction:**

Documenting Tunisian’ stress responses to an unprecedented pandemic is essential for mental health interventions and policy-making.

**Objectives:**

To describe the perceived stress generated by the Covid-19 epidemic and confinement among the Tunisian people.

**Methods:**

Participants had to fill out a questionnaire including epidemiological data and the Perceived Stress Scale 10 (PSS10), which is the most widely used psychological instrument for measuring the stress perception. Individual scores can range from 0 to 40 with higher scores indicating higher perceived stress.

**Results:**

Our study included 121 subjects, of which 70.6% were women.They had an average age of 36.52 years and a history of psychiatric disorders in 13.1% of cases, such as anxiety disorders (10.4%), depressive disorders (5.9%) and obsessive compulsive disorders (2.3%). More than one in two participants (61.4%) reported the presence of sleep disorders. Regarding medical history, participants declared having asthma (5%), diabetes (1.8%), high blood pressure (3.6%), and a chronic disease with corticosteroid treatment (5%). The mean PSS score was 16.96. This last was correlated to age (p<0.001), female gender (p<0.001), primary or secondary school level (p=0.03), a history of anxiety (p<0.001) and depressive disorders (p<0.001), and to sleep disorders (p<0.001).

**Conclusions:**

The stress level among the Tunisian people during the Covid-19 pandemic was very close to that observed in other countries, deserving special attention especially among vulnerable populations.

